# Supplementary material for: DeMBA: a developmental atlas for navigating the mouse brain in space and time
Source: Nat Commun. 2025 Aug 29;16:8108. doi: 10.1038/s41467-025-63177-9 (PMC12397216; doi:10.1038/s41467-025-63177-9)
Supplement: Supplementary file 1 — Description of Additional Supplementary Files [file 41467_2025_63177_MOESM1_ESM.pdf]

Description of Additional Supplementary Files

Supplementary Movie 1: Video showing a fly-through of the four-dimensional DeMBA template, starting with a volumetric rendering followed by section views through the three standard planes.

Supplementary Movie 2: Video showing a fly-through of the four-dimensional calbindin dataset, starting with a volumetric rendering followed by section views through the three standard planes.

Supplementary Data 1: Overview and description of all the anatomical landmarks used for validation of the DeMBA transformations.

Supplementary Data 2: Summary of statistics for the accuracy analysis presented in Figure 2.
